# FibroAtlas: A Database for the Exploration of Fibrotic Diseases and Their Genes

**DOI:** 10.1155/2019/4237285

**Published:** 2019-12-30

**Authors:** Jinying Liu, Dezhi Sun, Jiale Liu, Hao Xu, Yuan Liu, Yang Li, Lihong Diao, Xun Wang, Dan Wang, Lei Tian, Huimin Zhang, Zhongyang Liu, Weiquan Ren, Fuchu He, Dong Li, Shuzhen Guo

**Affiliations:** ^1^College of Traditional Chinese Medicine, Chengde Medical University, Chengde, Hebei Province, China; ^2^School of Traditional Chinese Medicine, Beijing University of Chinese Medicine, Beijing, China; ^3^State Key Laboratory of Proteomics, Beijing Proteome Research Center, National Center for Protein Sciences (PHOENIX Center), Beijing Institute of Lifeomics, Beijing, China

## Abstract

**Background:**

Fibrosis is a highly dynamic process caused by prolonged injury, deregulation of the normal processes of wound healing, and extensive deposition of extracellular matrix (ECM) proteins. During fibrosis process, multiple genes interact with environmental factors. Over recent decades, tons of fibrosis-related genes have been identified to shed light on the particular clinical manifestations of this complex process. However, the genetics information about fibrosis is dispersed in lots of extensive literature.

**Methods:**

We extracted data from literature abstracts in PubMed by text mining, and manually curated the literature and identified the evidence sentences.

**Results:**

We presented FibroAtlas, which included 1,439 well-annotated fibrosis-associated genes. FibroAtlas 1.0 is the first attempt to build a nonredundant and comprehensive catalog of fibrosis-related genes with supporting evidence derived from curated published literature and allows us to have an overview of human fibrosis-related genes.

## 1. Introduction

Fibrosis is a chronic and progressive process characterized by an excessive deposition of extracellular matrix (ECM) leading to overgrowth, hardening, and/or scarring of various tissues [1]. Fibrotic changes may affect almost all the main tissues and organs, including the skin, kidney, lung, and liver, as well as various vascular disorders [2]. Failure to control the abnormal wound healing responses can lead to considerable tissue remodeling and organ malfunction as seen in late-stage idiopathic pulmonary fibrosis and cardiac fibrosis [2, 3]. Aberrant fibrotic tissue remodeling also may be involved in the tumor initiation and progression, and accelerate chronic graft rejection in recipients of organ transplantation [4]. Fibrosis is one of the major causes of morbidity and mortality. Approximately 45 percent of all-cause mortality in the United States was attributed to fibrotic disorders [1].

Identification of effective therapeutic targets and designation for antifibrotic treatment strategies will depend on the underlying etiology, the severity, and extent of the fibrotic disease. However, the etiology and pathogenesis of fibrosis still remain virtually unknown, which limits our ability to optimally prevent or treat this disease. The natural history and the factors associated with fibrosis progression are highly variable [5]. Currently, lots of studies have indicated that both genetic factors and environmental exposures have been implicated in the formation and progression of fibrosis. For example, rs 35705950, a common polymorphism in the promoter of Mucin 5B (MUC5B), is associated with familial interstitial pneumonia and idiopathic pulmonary fibrosis, which suggests a crucial role of dysregulated MUC5B expression in the pathogenesis of pulmonary fibrosis [6]. Platelet factor 4 (PF4) is identified as a marker for fibrosis, levels of which are elevated in patients with systemic sclerosis and correlated with the presence and progression of pulmonary arterial hypertension [7]. Studies have suggested that multiple fibrotic diseases are usually triggered by the same irritation and share a number of common pathways, such as transforming growth factor beta (TGF-*β*), interleukin-6 (IL-6), and integrin-linked kinase signaling [8, 9].

Besides, there is still no database concentrating on fibrosis-associated genes. Therefore, a targeted strategy should be established to collect the magnanimity information about previously reported fibrosis-associated genes. To address the challenge, we create the FibroAtlas database 1.0 (http://biokb.ncpsb.org/fibroatlas/), which identifies 1,439 manual curated fibrosis-related genes by literature mining. FibroAtlas will shed light on the pathogenesis of individual cases, novel biomarkers for diagnosis and prognosis, and personalized therapeutic strategies.

## 2. Materials and Methods

### 2.1. Literature Mining and Manual Curation

We have constructed an ontology-based bioentity recognizer to recognize and extract genes in PubMed abstracts. This system compares favorably with current state-of-the-art biomedical annotation systems such as BeCAS [10] and has been evaluated against the CRAFT [11] corpus for gene/protein recognition based on Protein Ontology (PR) [12], which has the precision, F-measure, and recall of 0.959, 0.802, and 0.874, respectively. This system has been used to build AllerGAtlas 1.0 [13] successfully.

Three steps were taken to compile a comprehensive catalogue of human candidate genes related to fibrosis from PubMed abstracts.

First, 227,458 sentences in 114,973 PubMed abstracts including the keywords of “fibrosis,” “fibrotic,” “fibrotic action,” “fibrotic change,” or their lexical variants were identified by our bioentity recognizer.

Second, a list of 4,079 human genes with the fibrosis-associated keywords at sentence level co-occurrences were identified and extracted from 62,302 sentences in 10,243 PubMed abstracts by bioentity recognizer based on Protein Ontology (Supplementary material: [Supplementary-material supplementary-material-1].xlsx).

Third, 4,079 candidate genes were manually curated by our experts and 1,439 genes were finally certified as the human fibrosis-associated genes.

The co-occurrences between fibrosis-associated genes/proteins and fibrosis-related disease terminology based on Human Disease Ontology (DO) [14] were identified at sentence level from PubMed abstracts by bioentity recognizer. Furthermore, the genes identified as biomarkers were mined and marked with the terms “biomarker,” “biomarkers,” “marker,” “markers,” or “mark,” and then these potential biomarkers were manually curated by our experts.

### 2.2. Gene Annotation

We provided detailed annotations for each fibrosis-related gene to facilitate deeper interpretations for users. NCBI Entrez Gene ID and gene symbol were used for cross links and annotations. The basic gene information including gene symbol, synonyms, gene summary, chromosome, and chromosomal location were supplied to facilitate alignment known splicing sites. Gene ontology (GO) annotations were taken from the AmiGO database [15], and the gene-pathway relations were obtained from the Reactome database [16]. SNPs linked to genes were retrieved by the literature's PMIDs (PubMed Unique Identifier) from the dbSNP database [17]. The public databases such as Ensembl [18], Entrez gene [19], UniProt [20], neXtProt [21], and Antibodypedia [22] were also utilized to map and annotate.

## 3. Results

### 3.1. Database Implementation and Service

All identified fibrosis-related genes/proteins, human disease terminology, and their biomarkers were loaded into a local MySQL server. PHP was used to implement the web interface of FibroAtlas on a Windows server. All the data of FibroAtlas are accessible to every user without login or registration.

### 3.2. Database Search and Navigation

**Figure 1:**
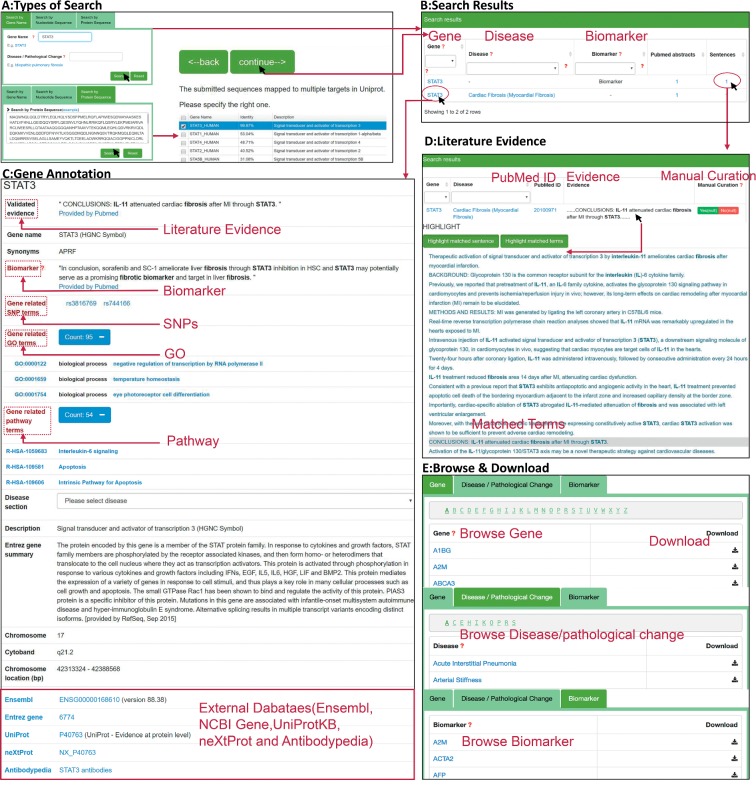
(A) Three main types of queries are supported by the “Home” page: gene symbol query, nucleotide sequence query, and protein sequence query. Users can input the gene symbol such as “STAT3” in the query box. Users can also input a nucleotide or protein sequence, and the sequence similarity identity score from BLAST will be displayed. Choose the matched gene name and click “continue” to scan the set of search results. (B) In the result page, a table including the queried gene, related disease terminology, and supporting evidences is listed. (C) By clicking the gene symbol of “STAT3” in the “search results” interface, users can browse detailed information of “STAT3” and cross links to external databases. (D) By clicking the number of PubMed abstracts or sentences in the “search results” interface, users can scan a table containing the information of gene, associated disease terminology, PubMed ID, evidence, and manual curation. Click the link of evidence in this page to scan the abstract with highlighted keywords. (E) Three approaches for browsing are presented in the “Browse & Download” page. All the data can be downloaded.

FibroAtlas is a user-friendly interface website to query the database (http://biokb.ncpsb.org/fibroatlas/), which has five components including “Home,” “Browse & Download,” “Feedback,” “FAQ,” and “Contact” (Figure 1). In the “Home” page, three main types of navigational queries are available: protein name, nucleotide sequence, and protein sequence. For example, if users submit a gene name in the search box of “Gene Symbol,” an autocompleted dropdown list of gene symbols will be displayed to show the possible matches in the FibroAtlas. Users can select one of them and click the “Search” button to jump to the result page. If users search the gene by nucleotide sequence or protein sequence, the sequence match scores from BLAST will be listed. Users can choose the matched gene name and click “continue” to browse result interface (Figure 1(A)). A table containing the queried gene, the supporting literature evidences for related human disease terminology, the role of gene, and the number of evidences will be displayed on the search result page by the search engine (Figure 1(B)). By clicking on the gene hyperlink, users can access the page of gene annotations, which includes a list of SNPs mapped to dbSNP, gene ontology (GO) terms derived from GOA, pathway identifiers derived from Reactome, and the gene description based on UniProtKB, etc. (Figure 1(C)). By clicking on the number of the evidence abstracts or sentences, users can browse a table containing the gene symbol, the PubMed ID, and the manual curated evidences. In addition, to specify individual interested evidence, users can obtain the whole abstract with highlighted names of entities, i.e., the alias names of gene and disease term (Figure 1(D)). Three approaches are supported by the page of “Browse & Download.” All the data can be freely downloaded (Figure 1(E)).

### 3.3. Application Case of the Database

**Figure 2:**
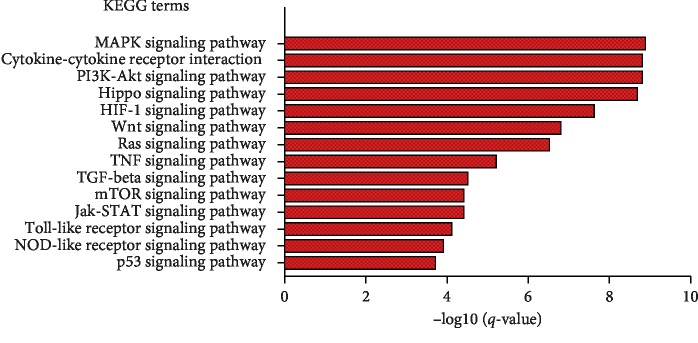
Bioinformatics pathway analysis for cardiac fibrosis-related gene sets with clusterProfiler [29].

Cardiac fibrosis is an inevitable consequence of chronic myocardial injury and leads to both systolic and diastolic dysfunction in many cardiac pathological conditions [23]. Cardiac fibrosis is a common phenomenon in the end stages of diverse cardiac diseases and is a predictive factor for sudden cardiac death [24]. There is an urgent need to unravel the intricate mechanisms underlying the development of cardiac fibrosis, in order to prevent long-term sequelae of cardiac fibrosis. We searched the database with the term of “cardiac fibrosis” and obtained 119 expert curated genes with detailed annotations. Pathway analyses were run on the list of cardiac fibrosis-related genes. The result shows that most of the genes share a number of common pathways and contribute in MAPK signaling pathway, cytokine-cytokine receptor interaction, Hippo signaling pathway, TGF-beta signaling pathway, and mTOR signaling pathway, etc (Figure 2). These results are validated by the literature and suggest that fibrosis arises as a consequence of multiple coactivated pathogenic pathways that affect inflammation and wound repair [25–27]. For example, yes-associated protein (Yap) acts as a transcriptional cofactor in the Hippo signaling pathway by activating the transcription of genes, inactivation of which after MI elicits increased myocyte apoptosis and fibrosis [28]. Furthermore, users can specify the hyperlink of the interested cardiac fibrosis-related genes to find the page with detailed functional annotation of genes, such as gene-related SNPs, pathways, and GO terms.

## 4. Discussion

**Figure 3:**
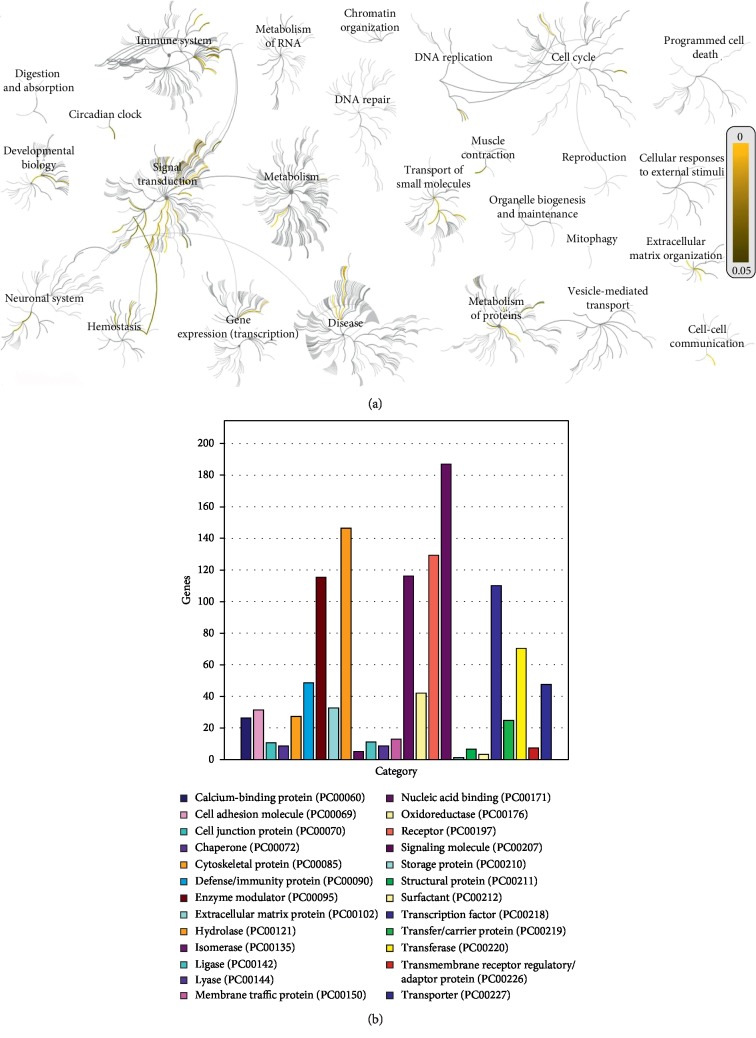
Bioinformatics analysis on the list of human fibrosis-related genes. (a) Biological pathway analysis with Reactome (http://www.reactome.org/). (b) Protein class analysis with PANTHER (http://pantherdb.org/).

Identification of key regulators of cell proliferation and quiescence is a significant step toward potential regenerative therapies [3, 30]. FibroAtlas 1.0 is the first complete and up-to-date gene network aiming to extract the literature on fibrosis-related genes and their function in diseases. FibroAtlas 1.0 (http://biokb.ncpsb.org/fibroatlas/), a powerful and time-saving tool with credible content, can provide accurate information and overview of human fibrosis-related genes. Analysis with Reactome (http://www.reactome.org/) [16] shows a strong tendency for these genes to participate in the pathways of signal transduction, immune system, cell cycle, hemostasis, gene expression (transcription), extracellular matrix organization, metabolism of proteins, developmental biology, neuronal system, cell-cell communication, transport of small molecules, muscle contraction, etc. (Figure 3(a)). The protein class analysis with DAVID (https://david.ncifcrf.gov) [31] reveals that these genes concentrate predominately on the role of signaling molecule, hydrolase, receptor, enzyme modulator, nucleic acid binding, defense/immunity protein, transcription factor, transferase, etc. (Figure 3(b)).

**Figure 4:**
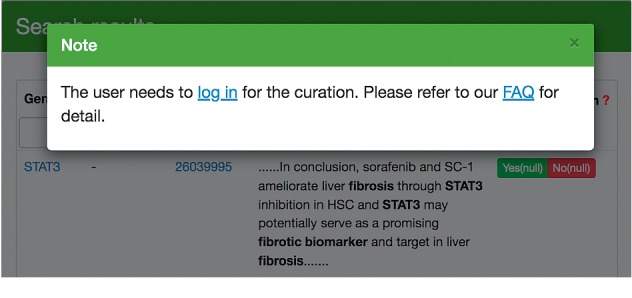
All logged-in users can give their feedback by clicking the “Yes” or “No” button to confirm or reject the evidence phrases.

A circulation system is supported by FibroAtlas 1.0. Sign in to give feedback by clicking the green “Yes” or red “No” button to accept or deny the evidence sentences (Figure 4). Our database will be periodically updated based on the results.

In future, we intend to carry out the following work to improve the performance of our database. Firstly, we will continue collecting fibrosis-related genes and replenishing genome-wide association studies data regularly. Second, we want to integrate the PPI information from both HPRD [32] and BioGRID [33] and then extract the direct interactors for fibrosis diseases candidate proteins in fibrosis-related genes. Finally, to help users to prioritize and select the information, we will further consider the following factors to implement a score for each fibrosis-related gene based on the supporting evidence, such as the number of supporting publications from text mining-based sources, the number of sources that report the association, the animal models and experimental strategies where the association has been studied, and the type of curation of each of these sources. In conclusion, we believe that FibroAtlas 1.0 will become a well-established resource with stable releases and be widely used as it can provide facilities for the research community and allied fields.

## Data Availability

The data sets generated during the current study are available in the FibroAtlas 1.0 repository (http://biokb.ncpsb.org/fibroatlas/).
